# The Self in Social Interactions: Sensory Attenuation of Auditory Action Effects Is Stronger in Interactions with Others

**DOI:** 10.1371/journal.pone.0022723

**Published:** 2011-07-27

**Authors:** Carmen Weiss, Arvid Herwig, Simone Schütz-Bosbach

**Affiliations:** 1 Max Planck Institute for Human Cognitive and Brain Sciences, Leipzig, Germany; 2 Department of Psychology, Bielefeld University, Bielefeld, Germany; Cuban Neuroscience Center, Cuba

## Abstract

The experience of oneself as an agent not only results from interactions with the inanimate environment, but often takes place in a social context. Interactions with other people have been suggested to play a key role in the construal of self-agency. Here, we investigated the influence of social interactions on sensory attenuation of action effects as a marker of pre-reflective self-agency. To this end, we compared the attenuation of the perceived loudness intensity of auditory action effects generated either by oneself or another person in either an individual, non-interactive or interactive action context. In line with previous research, the perceived loudness of self-generated sounds was attenuated compared to sounds generated by another person. Most importantly, this effect was strongly modulated by social interactions between self and other. Sensory attenuation of self- and other-generated sounds was increased in interactive as compared to the respective individual action contexts. This is the first experimental evidence suggesting that pre-reflective self-agency can extend to and is shaped by interactions between individuals.

## Introduction

The sense of generating and controlling our own actions and their perceptual consequences is suggested to contribute to a basic sense of selfhood [1], [2]. This experience of self-agency not only results from interactions with the inanimate environment (e.g., switching on a light), but also from interactions in social contexts. Our actions are intended to influence other people and, likewise, they are also influenced by others. Others' responsiveness to one's own behavior (e.g., smiling back) has been suggested to allow a reflection on and even the construal of self-agency [3]–[5]. Thus, the self is not just solipsistic in nature, but is thought to be especially organized to develop in interaction with others [6], [7]. This assumption, however, has not been experimentally addressed yet.

Here, we provide this first experimental approach to study the impact of social interactions on self-agency. We focused on sensory attenuation of action effects as a marker of pre-reflective self-agency [8], [9]. Sensory attenuation has been suggested to result from a comparison of the internally generated motor predictions about the sensory consequences of one's ongoing actions with the actual sensory consequences: If the two correspond, the sensory percept is attenuated, thereby enabling a differentiation between self-generated and externally generated sensory events [10], [11]. That is, on the perceptual level, a self-generated sensory stimulus is perceived as less intense than the same stimulus generated externally [12]–[15]. In the present study, we compared the attenuation of the perceived loudness intensity of auditory action effects generated via button press either by oneself or another person in either an individual, non-interactive or interactive action context. Following the notion of a social construal of self-agency, we predicted that social interactions between self and other would enhance the pre-reflective experience of self-agency as indicated via an enhancement of sensory attenuation.

## Methods

### Participants

Forty healthy participants (mean age 24.2 years, *SD* = 2.8 years, 22 female, all right-handed) with normal hearing (self report) took part in this study. Informed written consent was obtained from each participant prior to the experiment and they received a small reimbursement for their participation. The study was conducted in accordance with the Declaration of Helsinki and was approved by the local ethics committee of the University of Leipzig.

### Experimental procedure

The experiment was divided into two sessions completed on subsequent days. Each session comprised two parts: 1) an acquisition phase and 2) a test phase. The basic experimental protocol used to measure the loudness perception of sounds was adapted from [16].

#### 1) Acquisition phase

The first part consisted of two randomly ordered blocks with 200 trials each. In one block, participants performed voluntary button presses with their right index finger, followed by a standard tone of 74 dB 50 ms later. In another block, they observed the experimenter doing this. Thereby, an association between the button press action and its auditory consequence was build up for either agent.

#### 2) Test phase

**Figure 1:**
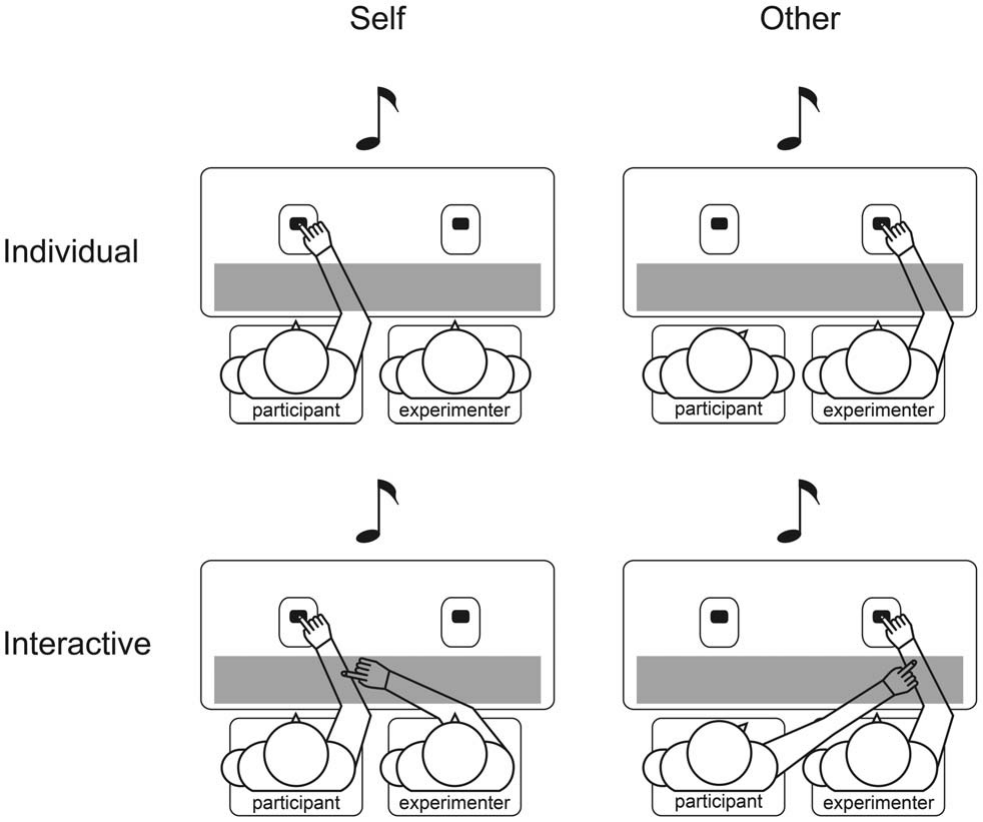
Experimental setup. Participants listened to tones that were either generated by their own button press (left column: Self) or another person's (i.e., experimenter) button press (right column: Other). One's own and the other's tone-eliciting button presses were either performed individually (upper row: Individual) or on request of the respective other person (lower row: Interactive).

The second part consisted of four experimental conditions (see Figure 1) that were presented as separate blocks. The four conditions were randomly subdivided across the two sessions; that is, participants performed two of them in the first session and the remaining two in the second session. The order and assignment of each condition to one of the two sessions was counterbalanced between participants.

Two of the conditions were individual conditions in which each agent performed his/her button press without being involved in any interaction with the other person. That is, participants performed a self-determined button press that generated the 74 dB standard tone 50 ms later or they observed the experimenter doing this. The other two conditions were interactive conditions. Here, participants performed the tone-eliciting button press whenever the experimenter requested them to do so by touching the participant's right forearm, which was occluded. Accordingly, the experimenter performed the tone-eliciting button press whenever the participant requested him to do so by touching the experimenter's right forearm, which was likewise occluded. Throughout the whole experiment, the participant and experimenter's hands were clearly visible while pressing the buttons, but their forearms were occluded to prevent visual anticipation of touch.

In each trial of the test phase, the 74 dB standard tone generated by the button press was followed by a comparison tone of varying magnitude (seven magnitude levels ranging from 71 and 77 dB in 1 dB steps) after a random interval of 800–1200 ms. Each comparison tone magnitude was presented 25 times per condition. All tone stimuli were sine tones of 1000 Hz lasting 100 ms and were presented binaurally through headphones. Participants always had to judge which of the two tones was louder. They gave their judgment by pressing one of two response keys labeled as “Tone 1” and “Tone 2”, respectively, with their left hand.

### Data analysis

After the experiment, the proportion of “second tone louder” responses was calculated for each participant and condition for the seven magnitudes of the comparison tone. Then, each set of “second tone louder” responses was fitted with a logistic function according to a maximum-likelihood procedure. Based on each individual function, the point of subjective equality (PSE), that is, the comparison tone magnitude judged as louder than the standard tone on 50% of trials, was obtained. Accordingly, the PSE value indicated the magnitude of the comparison tone that was perceived as equally loud as the magnitude of the standard tone.

## Results

**Figure 2:**
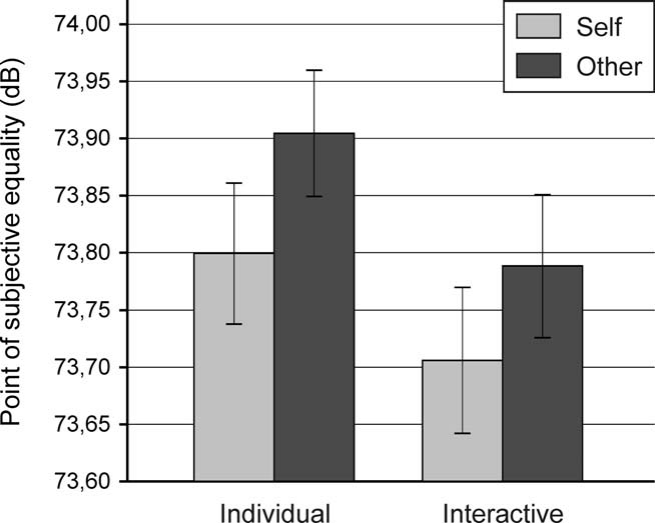
Mean and standard error of the point of subjective equality (dB) in the four experimental conditions.

The PSE values were entered into a repeated measures analysis of variance (ANOVA) with the two within-subject variables Button Press (self vs. other) and Action Context (individual vs. interactive). Figure 2 shows the PSE values in the resulting four experimental conditions. Both main effects were significant. The significant main effect of Button Press (*F*(1, 39) = 5.87, *p* = .020) indicated a lower PSE value – that is, stronger attenuation of the perceived loudness – for tones generated by one's own compared to the observed other's button presses. Most importantly, the significant main effect of Action Context (*F*(1, 39) = 9.64, *p* = .004) indicated a lower PSE value for tones generated by button presses in the interactive as opposed to the individual action context. Two planned comparisons confirmed this effect for both one's own button presses (*t*(39) = 1.79, *p* = .041) and the other's button presses (*t*(39) = 2.11, *p* = .021). In line with this, the main effects of Button Press and Action Context were not further qualified by an interaction between the two variables (*F*(1, 39) = 0.08, *p* = .785).

## Discussion

First of all, the present study confirms sensory attenuation of self-generated action effects [12]–[15]. Sounds generated by one's own button presses were perceived as less loud than sounds generated by another person's button presses (cf. [15]). Importantly, this functional signature of pre-reflective self-agency was strongly modulated by interactions between self and other.

Participants' perception of the loudness of a sound generated by another person was significantly reduced in the interactive action context, that is, when the other person was acting on request of the participant compared to when the other person generated them individually. We suggest that in the former case, the other person may become an integral part of one's own internal sensorimotor loop that then specifies the relation between one's own transmitting action, the other's responsive action and sensory consequence [17]. Thus, here, self-registration in action (effects) is a result of successfully making somebody else produce them.

The most intriguing finding is the stronger attenuation of sounds resulting from one's own button presses performed on request of the other person compared to one's own individually performed button presses. We speculate that this might be due to a kind of contrastive enhancement of self-agency in the interactive action context, where the causal initiation of one's button press was shifted towards an external source (i.e., the other person). This in turn necessitates and might facilitate access to other (internally) present cues which signal one's own agency contribution. Thus, internally generated motor predictions might receive a stronger weighting and therefore result in stronger sensory attenuation (cf. optimal cue integration for the experience of self-agency; [18]).

Overall, these results provide the first experimental evidence suggesting that pre-reflective self-agency is not exclusively determined intraindividually, but can extend to and is shaped by interactions between individuals. It remains to be investigated whether the interindividual element is a specific characteristic of human interactions or would also apply to similar “interactions” with non-human “agents” like robots.
